# Supplementary material for: De novo inter-regional coactivations of preconfigured local ensembles support memory
Source: Nat Commun. 2022 Mar 11;13:1272. doi: 10.1038/s41467-022-28929-x (PMC8917150; doi:10.1038/s41467-022-28929-x)
Supplement: Supplementary file 1 — Supplementary Information [file 41467_2022_28929_MOESM1_ESM.pdf]

Supplementary Information for

De novo inter-regional coactivations of preconfigured local ensembles support memory

Hiroyuki Miyawaki[1]*, Kenji Mizuseki[1]*

[1]Department of Physiology, Osaka City University Graduate School of Medicine,
Asahimachi 1-4-3, Abeno-ku, Osaka, 545-8585, Japan

*Correspondence: miyawaki.hiroyuki@med.osaka-cu.ac.jp (HM)
mizuseki.kenji@med.osaka-cu.ac.jp (KM)

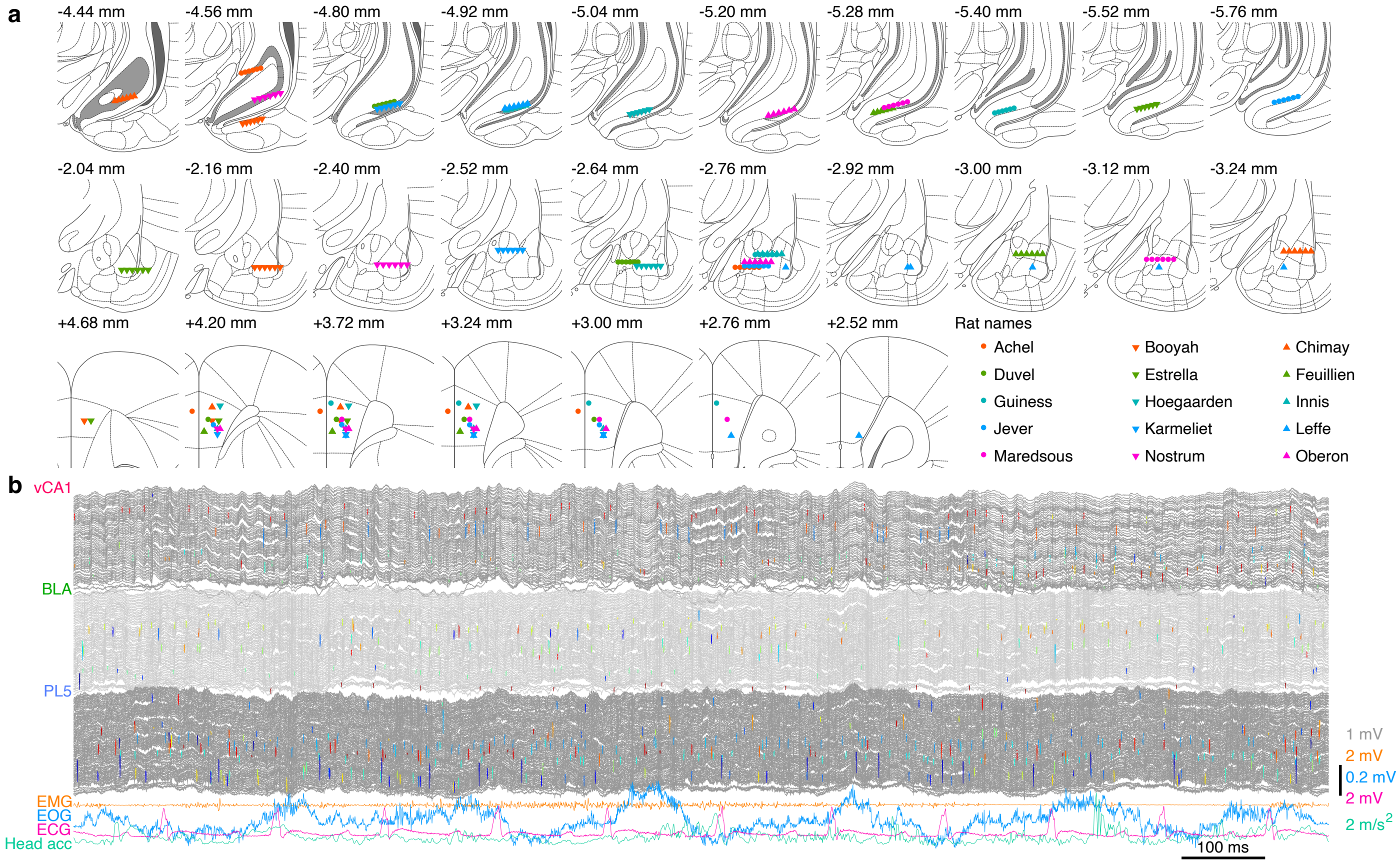

Supplementary Fig 1. Details of the electrophysiological recordings.

a Positions of probe tips of all rats marked on the coronal sections of the brain atlas adapted from a previous publication[65], with permission from Elsevier. The A-P axis coordinate from the bregma is shown at the top-left corner of each image.

b Example traces obtained from the rat shown in Fig. 1a, b. The raw traces recorded from the vCA1/BLA/PL5 are shown in grey, and well-isolated units are highlighted with colour. Different units are represented in different colours within each region. The four lower coloured traces represent the electromyography (EMG), electro-olfactography (EOG), electrocardiography (ECG), and head acceleration (Head acc).

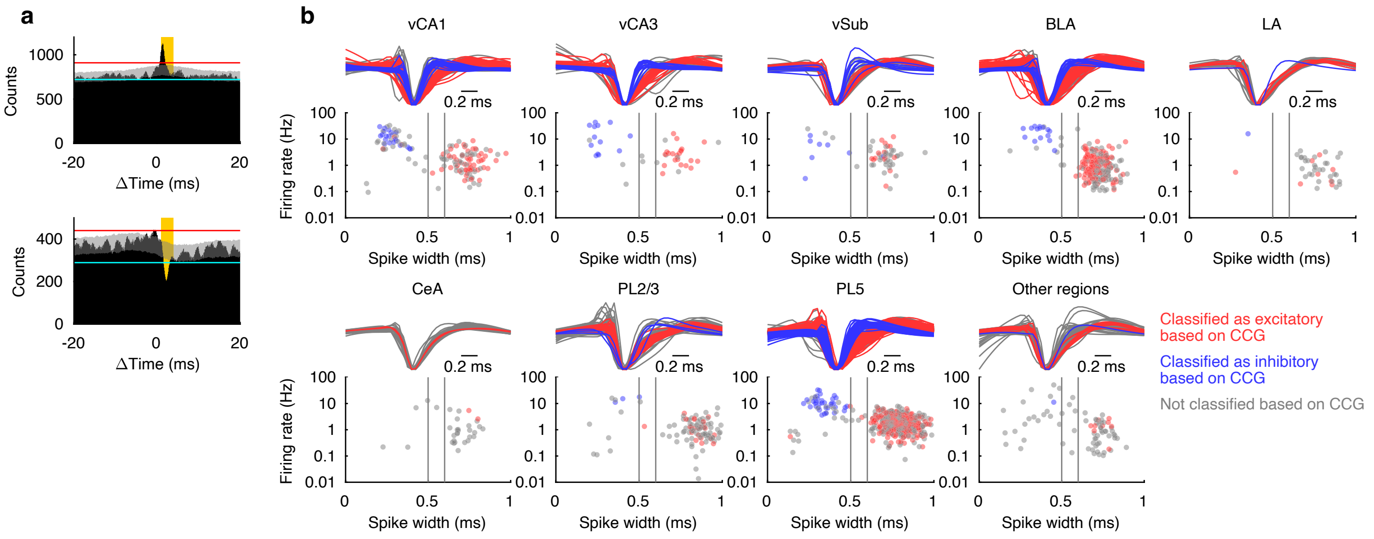

Supplementary Fig. 2 Excitatory and inhibitory cell classification.

a Example CCGs of spike times of neuron pairs with significant spike transmission (top) and suppression (bottom). The grey bands illustrate the 99% confidence interval (CI) of jittered CCG at each time point. The horizontal lines indicate the upper and lower global bands (99% CI of peak height/trough depth detected in a ±5 ms range). The orange background indicates a period of 1–4 ms, during which the significance of the peaks or troughs was evaluated.

b Mean waveforms normalised to the spike amplitudes (top) and scatter plots of spike width versus mean firing rate (bottom) of all recorded units. The colours of the traces/dots indicate the unit classification based on the CCG. The vertical lines on the scatter plots indicate thresholds for excitatory (0.6 ms) and inhibitory (0.5 ms) cells, respectively. The numbers of cells are summarised in Supplementary Table 1. Units with positive-dominant spike waveforms (3, 1, 1, 1, 3, 9, and 7 units in vCA1, BLA, LA, CeA, PL2/3, PL5, and other regions, respectively) are excluded from the plots. Source data are provided as a Source Data file.

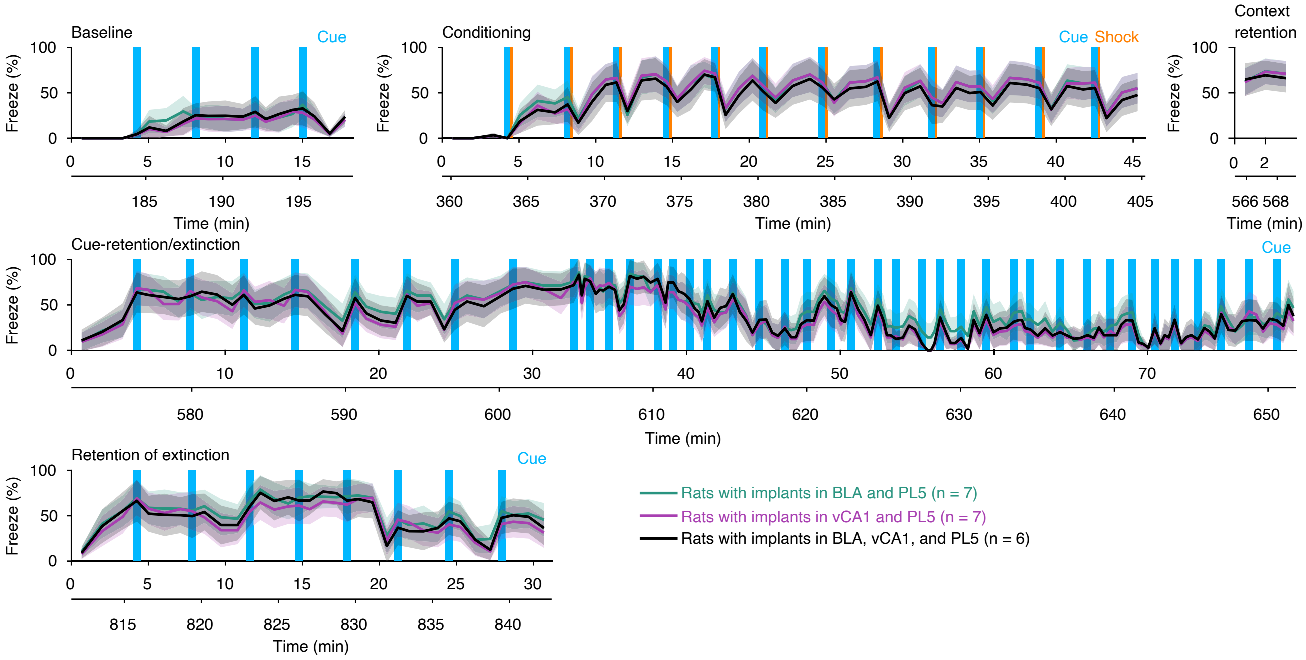

Supplementary Fig. 3 Summary of freezing behaviour for animal subsets with implants in specific brain regions.

Proportion of time spent in freezing behaviour, as shown in Fig. 1c, summarised for subsets of rats with implants in the BLA and PL5 (n = 7 rats, green); vCA1 and PL5 (n = 7 rats, purple); or BLA, vCA1, and PL5 (n = 6 rats, black). Lines and shaded areas indicate the means and standard errors of the mean, respectively. Source data are provided as a Source Data file.

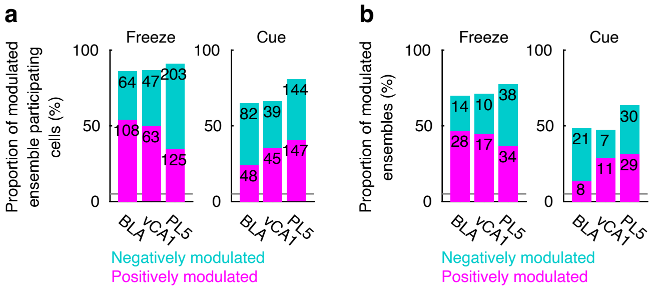

Supplementary Fig. 4 Cell firings and ensemble activations are modulated by freezing behaviour and cue presentation during cue-retention/extinction sessions.

Proportion of ensemble-participating-cells (cells that were members of at least one ensemble; **a**; n = 200/127/359 cells for BLA/vCA1/PL5, respectively) and proportion of ensembles (**b**; n = 60/38/93 for BLA/vCA1/PL5, respectively) whose firing rates/activation strength were significantly modulated by freezing behaviour (left) or cue presentation (right) during cue-retention/extinction sessions. The significance of the modulation of each cell firing/ensemble activation was determined by comparing the actual modulation strength with the distribution of modulation strengths in surrogates obtained using bin label shuffling (refer to Methods for detailed information). Horizontal bars indicate the chance level (5%). The numbers of cells and ensembles are superimposed on the bars. Source data are provided as a Source Data file.

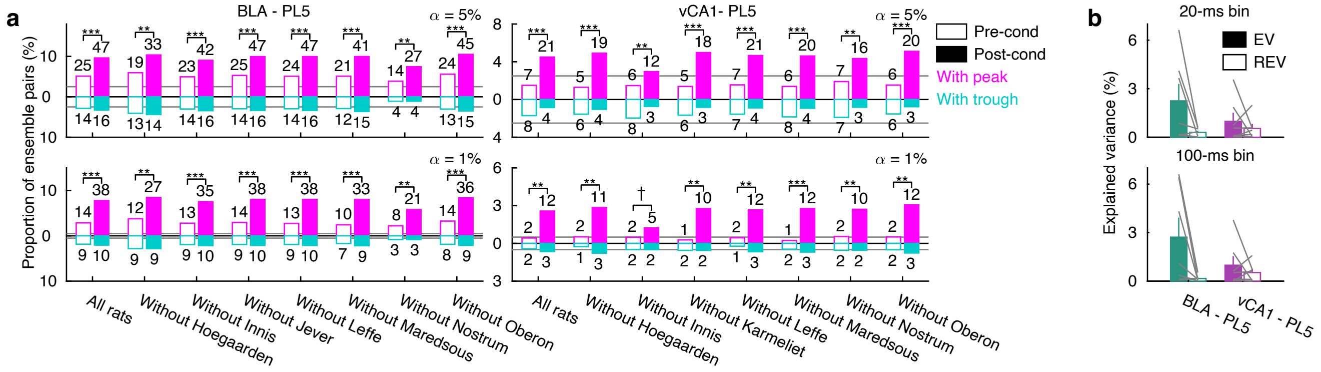

Supplementary Fig. 5 Consistency of coactivation enhancement in post-conditioning NREM across animals and analysis methods.

a Leave-one-out analysis of the proportion of coupled- and inverse-coupled-ensemble-pairs between pre-conditioning (pre-cond) and post-conditioning (post-cond) NREM. Hoegaarden, Innis, Jever, Leffe, Maredsous, Nostrum, and Oberon refer to the names of individual rats (see Supplementary Fig. 1). The top and bottom panels show the proportions that met the 95- and 99-percentile criteria, respectively. Horizontal bars indicate the chance level (2.5% and 0.5% for top and bottom panels, respectively). The numbers of ensemble pairs are superimposed on the bars. *** $p < 0.001$, ** $p < 0.01$, † $p = 0.06$, Fisher's exact test (including non-coupled pairs). Note that the 95-percentile criterion was used only for the top portion of this panel.

b Explained variance (EV) between spike count correlations in conditioning sessions and post-conditioning NREM under the control of correlation in pre-conditioning sessions and their reverse EV (REV) for BLA–PL5 and vCA1–PL5 cell pairs (n = 7 rats for each). The top and bottom panels show results with 20 and 100 ms bins, respectively. EV and REV obtained from each animal are connected by a grey line. The error bars indicate the standard errors of the mean.

Detailed statistics are shown in Supplementary Data 1, and source data are provided as a Source Data file.

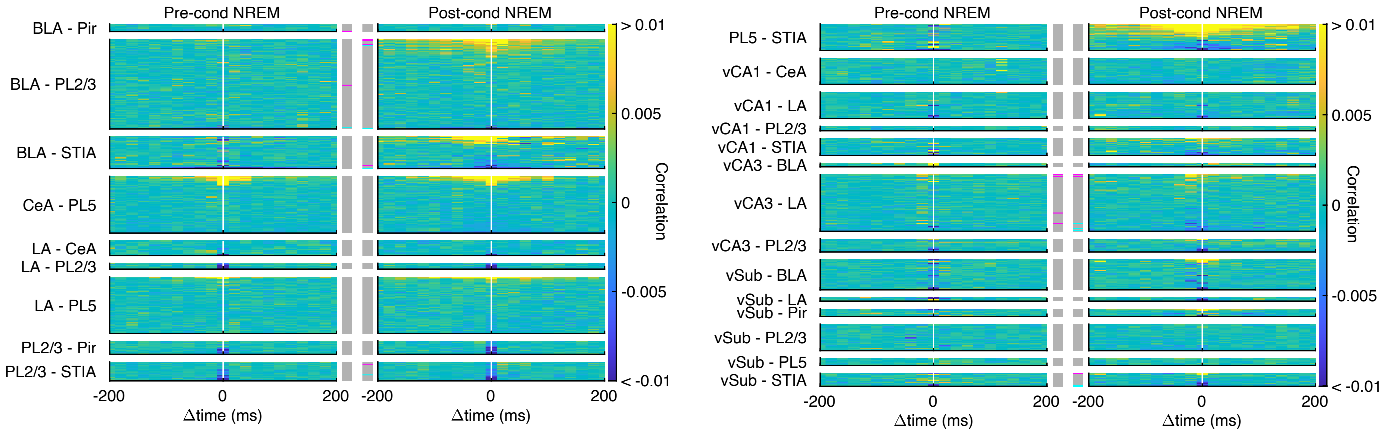

Supplementary Fig. 6 Coactivation of ensembles among various brain regions.

Inter-regional CCGs of the instantaneous activation strength of ensembles identified in conditioning sessions during the pre- and post-conditioning (cond) NREM. The coloured bars in the middle columns indicate ensemble pairs with significant peaks (magenta) and troughs (cyan), respectively ($p < 0.01$, chunk shuffling). The panels illustrate all analysed pairs other than the pairs formed among vCA1/BLA/PL5, shown in Fig. 3a. CeA, central nucleus of the amygdala; LA, lateral nucleus of the amygdala; Pir, pyriform cortex; PL2/3, prelimbic cortex layer 2/3; STIA, bed nucleus of the stria terminalis intra-amygdaloid division; vCA3, ventral hippocampus CA3 region; vSub, ventral subiculum. The numbers of analysed pairs are summarised in Supplementary Table 3. Source data are provided as a Source Data file.

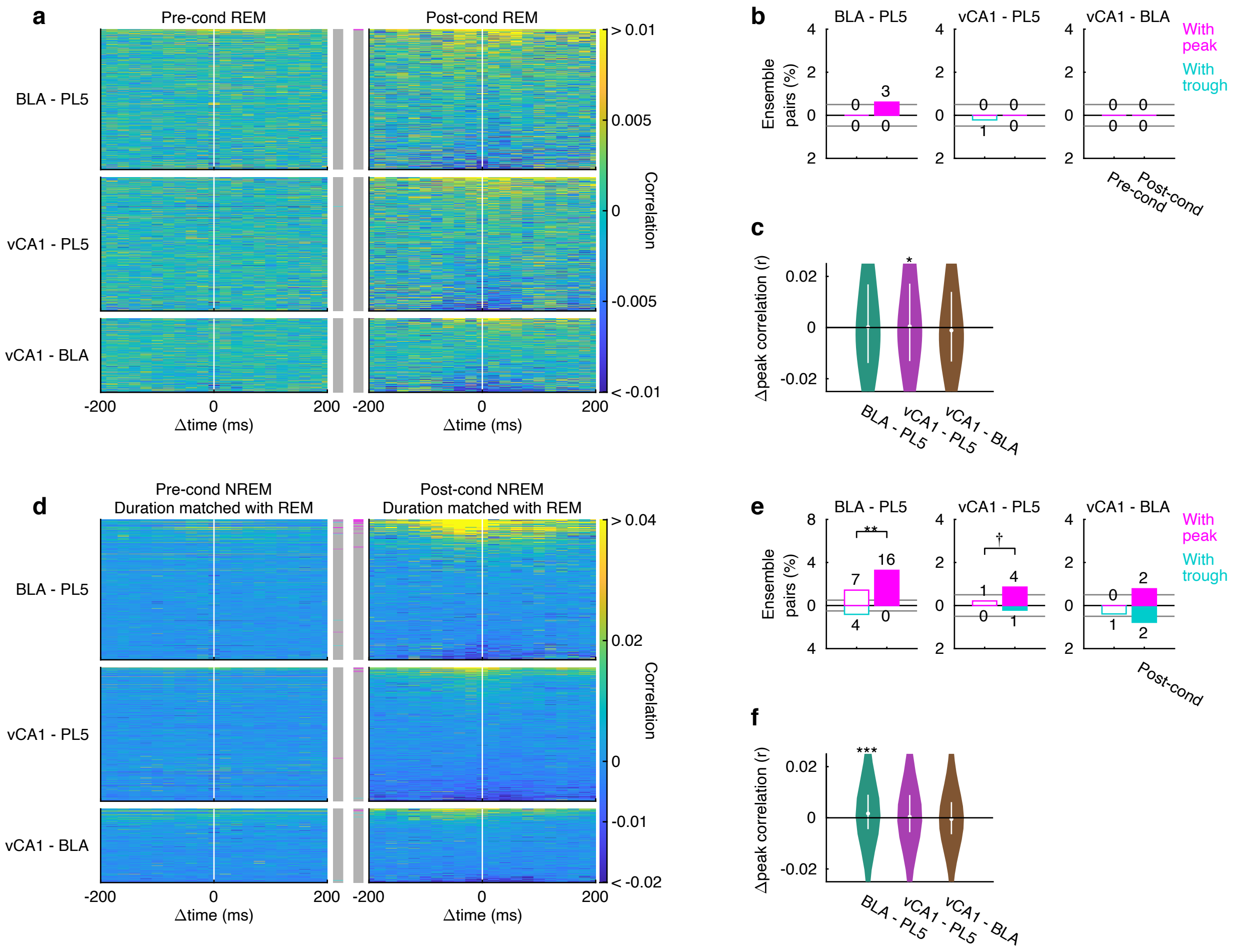

Supplementary Fig. 7 Inter-regional ensemble coactivation during REM.

a CCGs of the instantaneous activation strength of the ensembles identified in conditioning sessions during pre- and post-conditioning (cond) REM sleep.

b Proportion of ensemble pairs with significant peaks or troughs of CCGs shown in (**a**). Horizontal bars indicate chance levels (0.5% for each direction). The numbers of ensemble pairs are superimposed on the bars. No significant changes were detected ($p > 0.1$, Fisher's exact test).

c Violin plots indicating the changes in CCGs peak heights from pre- to post-cond REM for all ensemble pairs shown in (**a**). White dots and lines represent median and upper/lower quartiles, respectively. * $p < 0.05$, WSR-test.

d-f CCGs of instantaneous activation strength during pre- and post-conditioning (cond) NREM sleep, as shown in Fig. 3a-c, with the analysis duration matching that of REM sleep. For each rat, we matched the analysed durations of pre- and post-cond NREM with those of pre- and post-cond REM, respectively, using the beginning part of the concatenated NREM epochs whose duration was matched the total duration of REM epochs in the homecage session of interest. In (**f**), white dots and lines represent median and upper/lower quartiles, respectively. ** $p < 0.01$, † $p = 0.078$, Fisher's exact test in (**e**). *** $p < 0.001$ WSR-test in (**f**).

The numbers of ensemble pairs are summarised in Supplementary Table 4. Detailed statistics are shown in Supplementary Data 1, and source data are provided as a Source Data file.

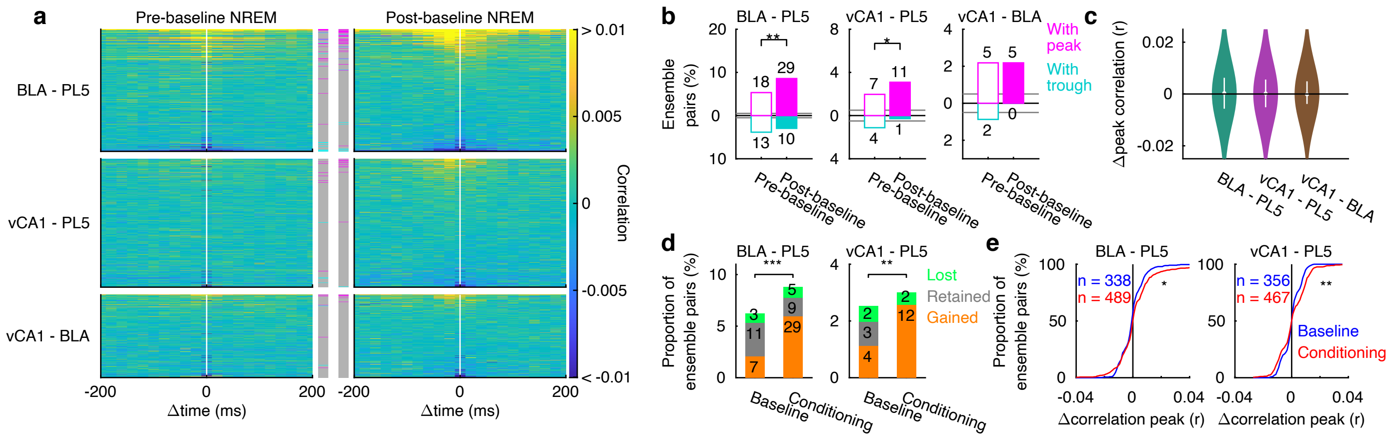

Supplementary Fig. 8 Conditioning sessions enhance coactivation to a greater extent than baseline sessions.

a–c CCGs of the instantaneous activation strength of the ensembles identified in the baseline sessions during NREM in homecage sessions preceding (pre-baseline) and following (post-baseline) the baseline sessions (**a**), proportion of ensemble pairs with significant peaks or troughs (**b**), and violin plots of changes in peak height (**c**) of the CCGs shown in (**a**) (n = 338, 356, 229 pairs for BLA–PL5, vCA1–PL5, and vCA1–BLA, respectively). In (**c**), white dots and lines represent median and upper/lower quartiles, respectively. ** $p < 0.01$, * $p < 0.05$, Fisher's exact test.

d Proportion of ensemble pairs that gained, retained, and lost significant CCGs peaks from pre- to post-baseline NREM and from pre- to post-conditioning NREM (n = 338/489, and 356/467, pairs for the BLA–PL5 and vCA1–PL5 groups, respectively). *** $p < 0.001$, ** $p < 0.01$, Fisher's exact test (including non-coupled pairs for both periods). The number of ensemble pairs is superimposed on the bars.

e Cumulative histogram of CCG peak change induced by baseline and conditioning sessions. The number of examined pairs is superimposed on the top left of each plot. ** $p < 0.01$, * $p < 0.05$, Kolmogorov–Smirnov test.

Detailed statistics are shown in Supplementary Data 1, and source data are provided as a Source Data file.

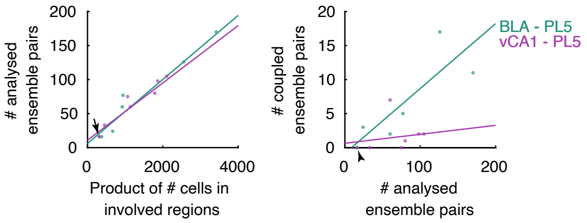

Supplementary Fig. 9 Association between the number of coupled-ensemble-pairs, the number of analysed ensemble pairs, and the product of the numbers of cells in involved regions.

Scatter plots of the product of the numbers of recorded cells versus the number of analysed ensemble pairs (left) and the number of analysed ensemble pairs versus the number of coupled-ensemble-pairs (right) for each rat (n = 7 rats for each panel). The arrow on the left panel and the arrowhead on the right panel represent two (one each from BLA–PL5 and vCA1–PL5) and three (two from BLA–PL5 and one from vCA1–PL5) data points overlapped on the same point, respectively. Regression lines are shown for BLA–PL5 and vCA1–PL5 pairs separately. Source data are provided as a Source Data file.

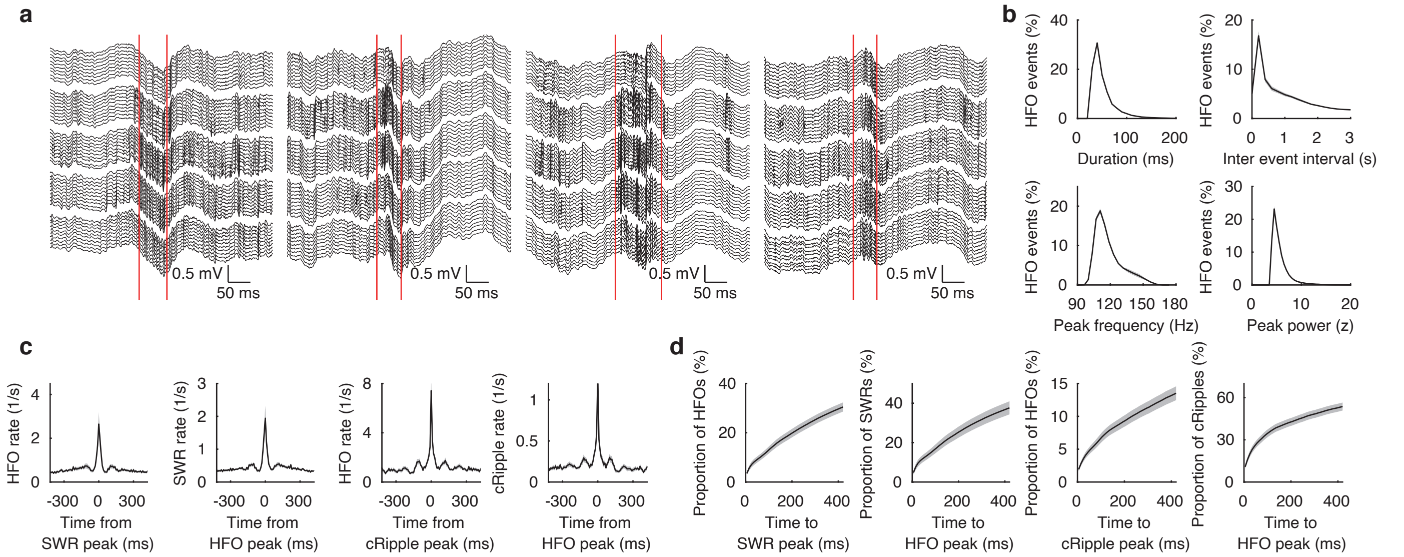

Supplementary Fig. 10 Amygdalar high-frequency oscillations.

a Representative examples of amygdalar HFOs. The onset and offset of each event are marked by red vertical lines.

b Histograms of duration, inter-event interval, peak frequency, and peak power of HFOs. The lines and grey shaded areas indicate the means and standard errors of the mean (SEMs; n = 15 rats), respectively.

c CCGs of HFOs versus other oscillatory events. The CCGs between HFO and SWR/cRipple events exhibit a noticeable peak at time zero. The lines and grey shaded areas indicate the means and SEMs, respectively (n = 14 rats for left 2 plots and n = 15 rats for right 2 plots).

d Cumulative histograms of intervals from HFO peaks to the closest SWR peaks (left), from SWR peaks to the closest HFO peaks (second left), from HFO peaks to the closest cRipple peaks (second right), and from cRipple peaks to the closest HFO peaks (right). The lines and grey shaded areas indicate the means and SEMs, respectively (n = 14 rats for left 2 plots and n = 15 rats for right 2 plots). Note that 12.6% ± 1.4% [mean ± SEM, n = 14 rats] and 6.5% ± 0.69% [mean ± SEM, n = 15 rats] of HFO peaks were detected within ±100 ms periods of SWR and cRipple peaks, respectively.

Source data are provided as a Source Data file.

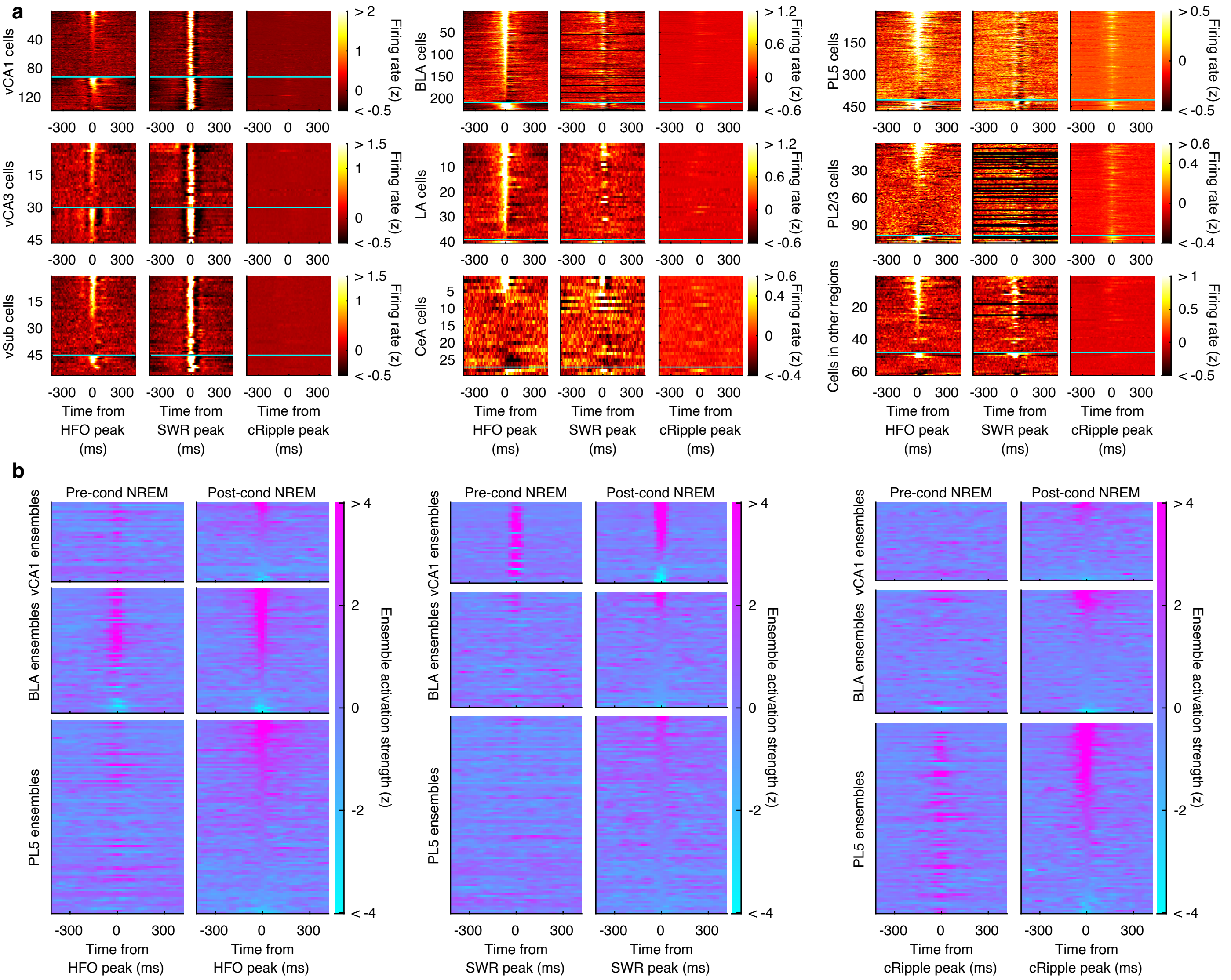

Supplementary Fig. 11 Cell firings and ensemble activations are modulated by fast oscillations.

a HFO-, SWR-, and cRipple-peak-triggered histograms of firing in each brain region. Each row indicates one cell. Cells plotted above the horizontal cyan lines are excitatory cells, while the others are inhibitory cells. Cells are ordered based on peak height at time zero in the HFO-triggered histograms. The numbers of cells are summarised in Supplementary Table 1.

b HFO-, SWR-, and cRipple-peak-triggered histograms of the instantaneous ensemble activation strength in the pre- and post-conditioning (cond) NREM. Ensembles are ordered based on peak height at time zero in the post-cond sessions for each trigger type. The numbers of ensembles are summarised in Supplementary Table 2.

Source data are provided as a Source Data file.

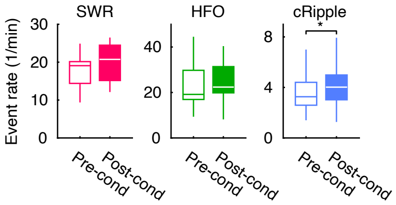

Supplementary Fig. 12 Occurrence rates of fast network oscillations in pre- and post-conditioning NREM.

Box plots indicating the SWR, HFO, and cRipple occurrence rates during the pre- and post-conditioning (cond) NREM. * $p < 0.05$, WSR-test ($n = 14$, 15, and 15 rats for SWRs, HFOs, and cRipples, respectively). Centre lines and limits of the boxes represent median and upper/lower quartiles, respectively, and whiskers extend to the maxima and minima with outliers excluded. Detailed statistics are shown in Supplementary Data 1, and source data are provided as a Source Data file.

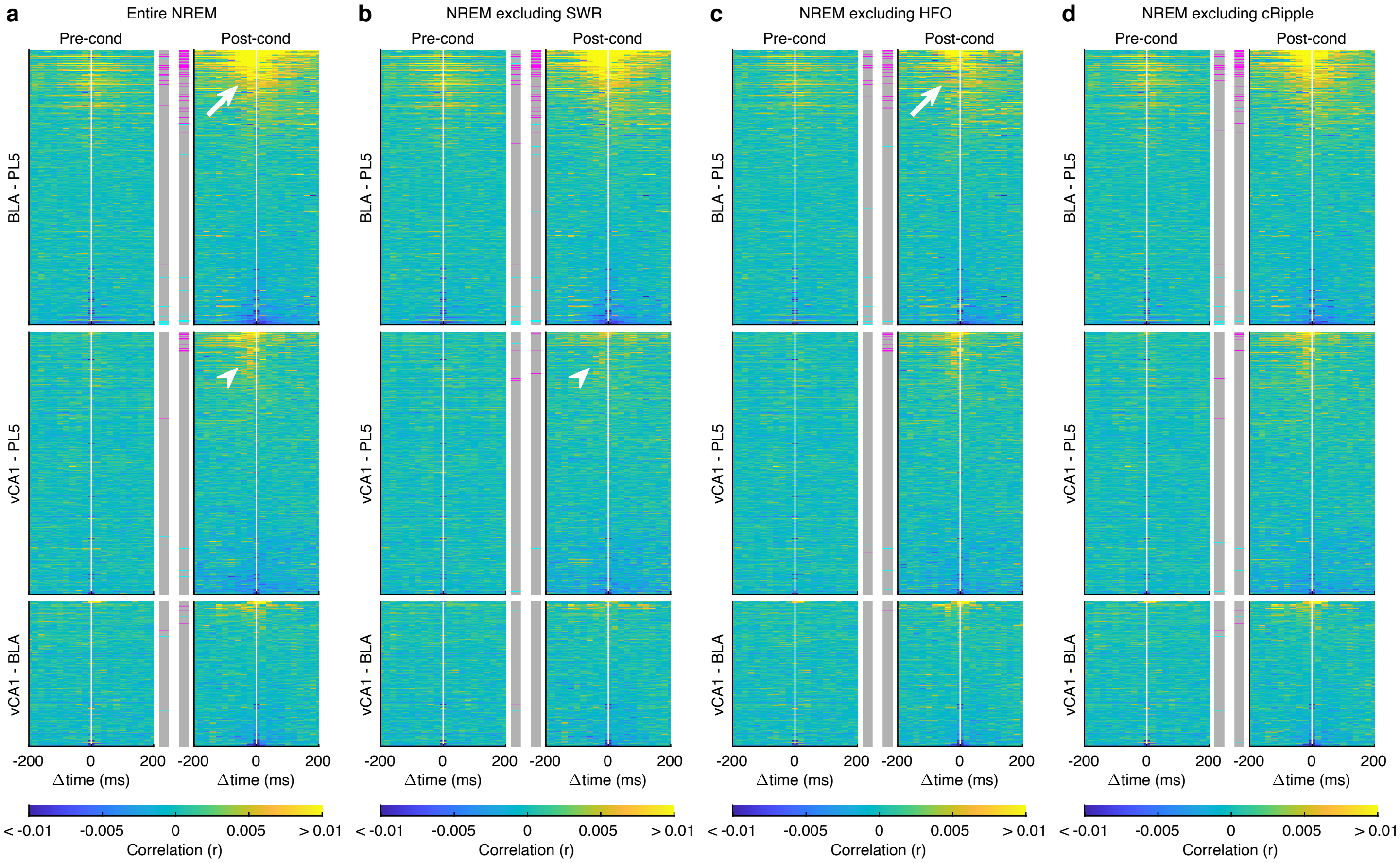

Supplementary Fig. 13 Inter-regional coactivation outside fast network oscillations.

CCGs of the instantaneous ensemble activation strength in the entire NREM (**a**), NREM outside SWRs (**b**), NREM outside HFOs (**c**), and NREM outside cRipples (**d**). CCGs in the entire NREM (**a**) are identical to those in Fig. 3a and are presented here for comparison. Ensemble pairs are sorted based on the peak height of the post-conditioning (cond) entire NREM CCG. The white arrows/arrowheads indicate CCG peaks between BLA–PL5/vCA1–PL5 ensemble pairs, which are less prominent on HFO-/SWR-excluded CCGs, respectively. The numbers of analysed pairs are summarised in Supplementary Table 4. Source data are provided as a Source Data file.

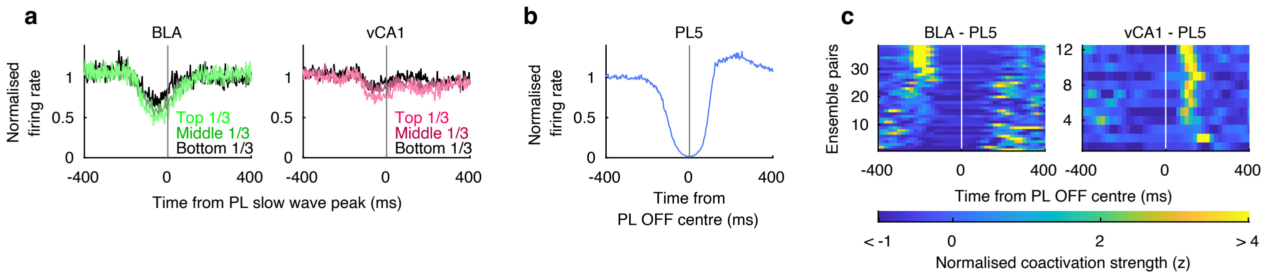

Supplementary Fig. 14 Ensemble coactivations are modulated by PL OFF state.

a PL slow-wave peak-triggered average firing rates of BLA (n = 12 rats) and vCA1 (n = 8 rats) neurons. Slow-waves were classified into tertiles based on their amplitudes within each rat, and the firing rates were normalised to the mean in the periods of [–2,000 ms, –1,500 ms] and [+1,500 ms, +2,000 ms] from the slow-wave peaks.

b PL OFF state centre-triggered average firing rates in PL5 (n = 8 rats). Firing rates were normalised to the mean in the periods of [–2,000 ms, –1,500 ms] and [+1,500 ms, +2,000 ms] from the OFF state centre.

c The PL OFF state centre-triggered average coactivation strengths of BLA–PL5 and vCA1–PL5 coupled-ensemble-pairs. Coactivation strength was z-scored in the periods of [–2,000 ms, +2,000 ms]. The numbers of analysed pairs are summarised in Supplementary Table 4.

Source data are provided as a Source Data file.

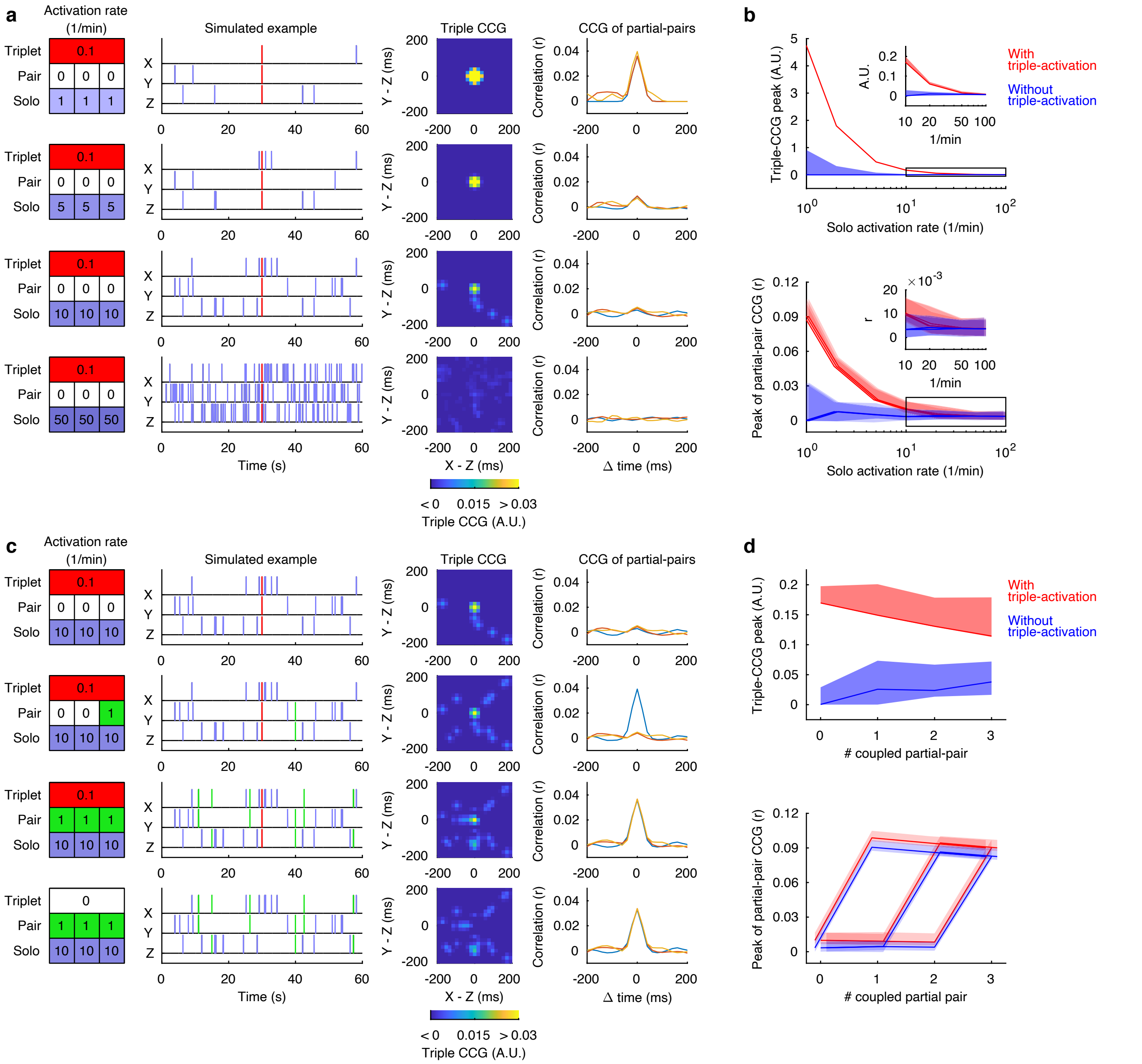

Supplementary Fig. 15 Effects of triple-activation, coactivation, and solo activation on triple CCG and CCGs of partial-pairs.

a, c Examples of triple CCG analysis on artificially generated data. Simulation settings (left column), first min of example signal trains (second column), triple CCG, and CCGs of partial-pairs of the example trains (right two columns) are shown. In the second column, triple-activation, pairwise coactivation, and solo activation events are highlighted in red, green, and blue, respectively.

b, d Peak values of triple CCGs (top) and CCGs of partial-pairs (bottom) with and without triple-activation are shown as a function of solo activation event rates (**b**) or as a function of the number of coupled partial-pairs (**d**). The triple-activation rate was fixed to 0.1/min (with triple-activation) or 0/min (without triple-activation) in both (**b**) and (**d**). The pairwise coactivation rate of partial-pairs was fixed to 0/min in (**b**) and 0/min (without pairwise coactivation) or 1/min (with pairwise coactivation) in (**d**). Coloured lines and shaded areas indicate median and 99% confidence interval, respectively (n = 500 simulations for each condition). In the bottom plots, small horizontal jitter is added for visibility. In (**b**), the magnification view of the region indicated with black rectangles is superimposed on the top right of each plot.

Source data are provided as a Source Data file.

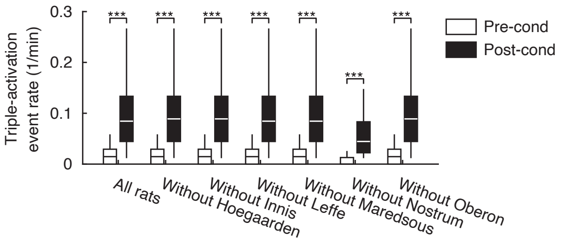

Supplementary Fig. 16 Triple-activation events are consistently enhanced across animals in the post-conditioning NREM.

Leave-one-out analysis of triple-activation event rate changes from pre-conditioning (pre-cond) to post-conditioning (post-cond) NREM, which confirm the robustness of the triple-activation enhancement across animals (n = 100, 84, 93, 98, 97, 36, and 92 triplets). Hoegaarden, Innis, Leffe, Maredsous, Nostrum, and Oberon refer to the names of individual rats (see Supplementary Fig. 1). Centre lines and limits of the boxes represent median and upper/lower quartiles, respectively, and whiskers extend to the maxima and minima with outliers excluded. *** $p < 0.001$, Wilcoxon signed-rank test. Detailed statistics are shown in Supplementary Data 1, and source data are provided as a Source Data file.

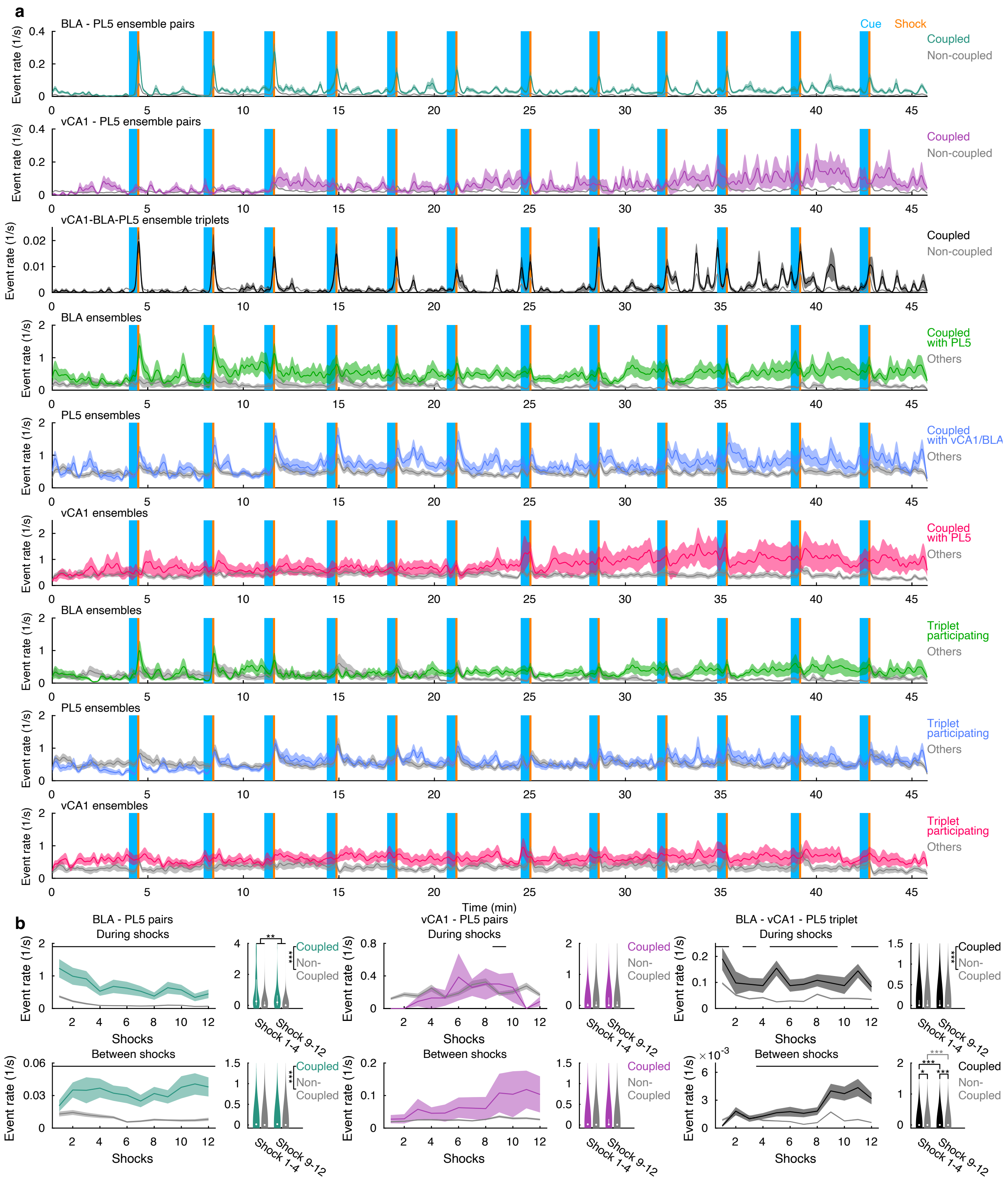

Supplementary Fig. 17 Time evolution of ensemble coactivations and triple activations by fear-conditioning.

a Mean coactivation rates of ensemble pairs (top two rows), triple-activation rates of ensemble triplets (third row), and activation event rates of ensembles (bottom six rows) during conditioning sessions. In the top three panels, coloured/black and grey lines indicate coupled and other (non-coupled) ensemble pairs/triplets, respectively. In the middle/bottom three panels, coloured lines show event rates of coactivation-/triplet-participating-ensembles, and grey lines illustrate mean event rates of the remaining ensembles (others). Shaded areas indicate standard errors of the mean (SEMs). Means and SEMs were calculated with 0.1 s bins and Gaussian smoothed (σ = 5 s).

b Mean coactivation (left two columns) and triple-activation (right column) event rates during (top row) or between (10–180 s after shock offset; bottom row) shock presentations. Averages for the first and last four shocks are summarised on the right panels. Shaded areas on the left panels indicate SEMs. White dots and lines on the right panels represent median and upper/lower quartiles, respectively. Black ticks on the left panels indicate shocks with a significant difference between coupled- and non-coupled-ensemble-pairs/triplets ($p < 0.05$, WRS-test). *** $p < 0.001$, ** $p < 0.01$, * $p < 0.05$, post-hoc TK test following two-way analysis of variance on the ranks.

The numbers of analysed ensemble pairs/triplets are summarised in Supplementary Tables 4, and those of ensembles are summarised in Supplementary Tables 5. Detailed statistics are shown in Supplementary Data 1, and source data are provided as a Source Data file.

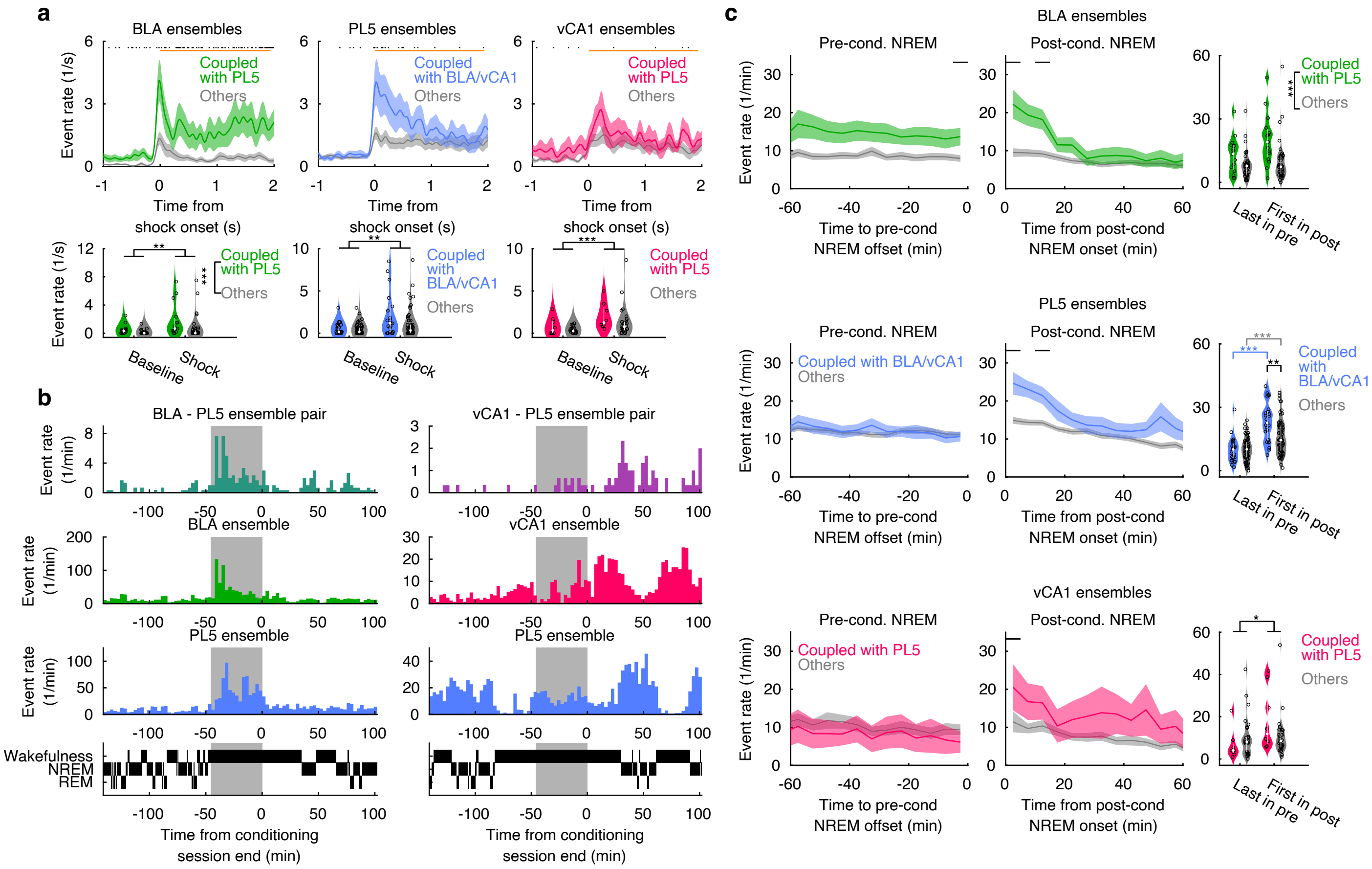

Supplementary Fig. 18 Ensemble activation time course.

a Shock-triggered average of the ensemble activation event rates. Top panels show the means of the occurrence rates, where lines and shaded areas represent means and standard errors of the mean (SEMs), respectively. Periods with a significant difference between coactivation-participating-ensembles (ensemble participating in at least one coupled-ensemble-pair among BLA, PL5, and vCA1) and other ensembles are indicated with black ticks on the top ($p < 0.05$, WSR-test). Bottom violin plots show event rates in periods 0.05–0.55 s prior to and following shock onsets (baseline and shock, respectively). White dots and lines represent median and upper/lower quartiles, respectively. Open circles indicate individual data points with random horizontal jitters. *** $p < 0.001$, ** $p < 0.01$, * $p < 0.05$, post-hoc Tukey–Kramer (TK) test following two-way analyses of variance (ANOVA) on the ranks.

b Representative examples of the time evolution in ensemble coactivations of BLA–PL5 and vCA1–PL5 coupled-ensemble-pairs (top row) and activations of ensembles consisting of the pair (middle two rows). The event rates were plotted regardless of the brain state. The grey backgrounds indicate periods of conditioning sessions. Hypnograms are shown at the bottom of the plot.

c Mean occurrence rates of ensemble activation events during NREM aligned to the offset/onset of the last/first NREM epochs in pre-/post-conditioning (cond) homecage sessions (left and middle) and violin plots indicating ensemble activation event rates during the last/first NREM in pre-/post-cond homecage sessions (right). For time aligned analysis (left and middle), all NREM epochs in pre/post-cond homecage sessions were concatenated. On the left and middle panels, shaded areas represent SEMs, and black ticks on the top indicate periods with significant differences between coactivation-participating-ensembles and other ensembles ($p < 0.05$, Wilcoxon signed-rank test). On the right panels, white dots and lines represent median and upper/lower quartiles, respectively. Open circles on the violin plots indicate individual data points with random horizontal jitters. *** $p < 0.001$, ** $p < 0.01$, * $p < 0.05$, post-hoc TK test following two-way ANOVA on the ranks.

Ensembles were detected based on neuronal firings during entire conditioning sessions and coupled regions of coactivation-participating-ensembles were determined in post-conditioning NREM. The numbers of analysed ensembles in (**a**) and (**c**) are summarised in Supplementary Table 5. Detailed statistics are shown in Supplementary Data 1, and source data are provided as a Source Data file.

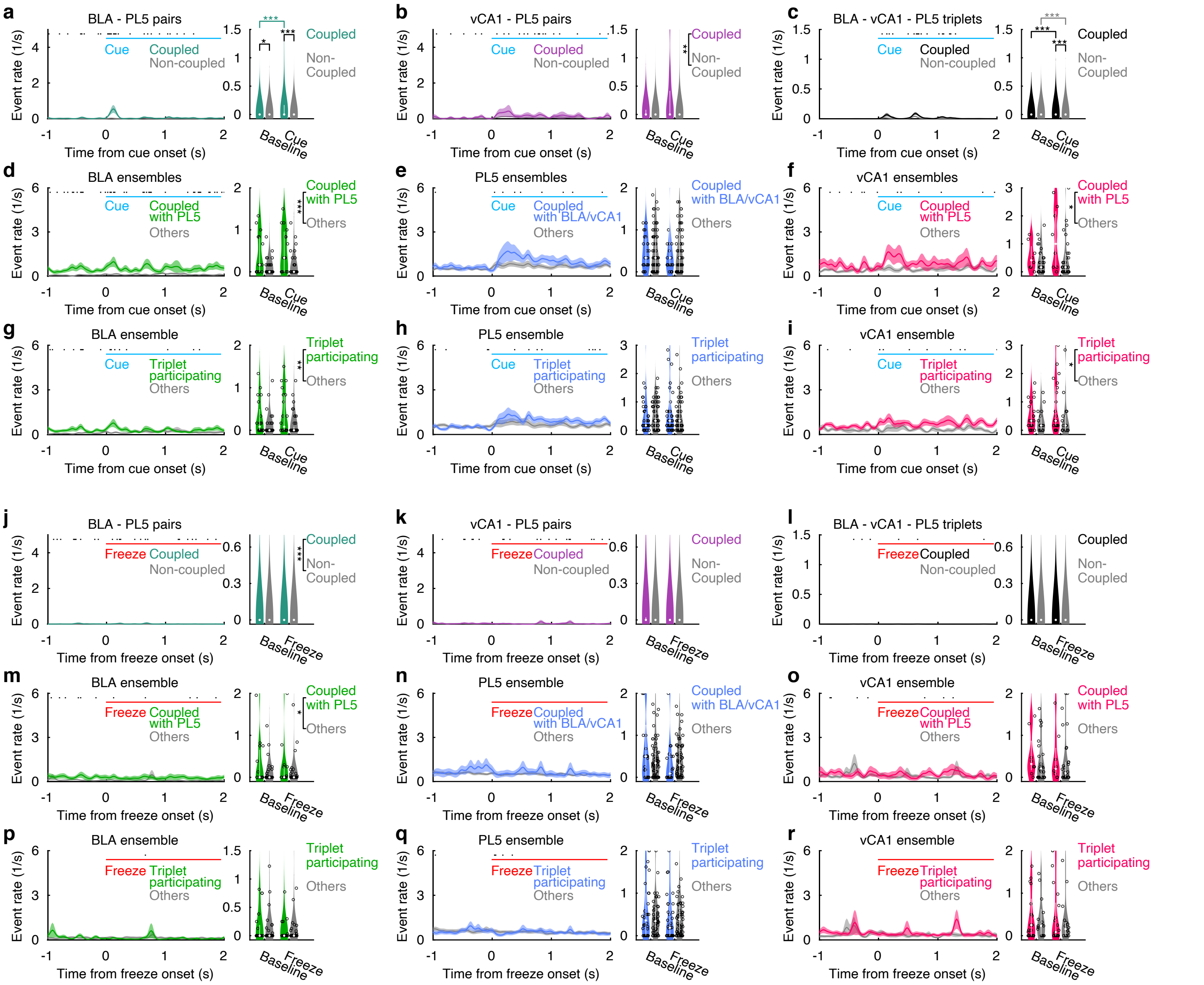

Supplementary Fig. 19 Cue and freeze onset-triggered average of ensemble activation, coactivation, and triple-activation event rates during conditioning sessions.

Cue (**a-i**) and freeze (**j-r**) onset-triggered average of coactivation/triple-activation/activation event rates during conditioning sessions. Violin plots on the right indicate means in periods of 0.05–0.55 s prior to and following the triggers (baseline and cue/freeze, respectively). Coloured/grey lines indicate means of coupled/non-coupled-ensemble-pairs (**a, b, j, k**), coupled/non-coupled triplets (**c, l**), coactivation-participating/other ensembles (**d-f, m-o**), and triplet-participating/other ensembles (**g-i, p-r**), respectively. The shaded areas on the left panels represent standard errors of the mean. On the right panels, white dots and lines represent medians and upper/lower quartiles. Open circles in the right panels of (**d-i**) and (**m-r**) show the event rate of each ensemble. *** $p < 0.001$, ** $p < 0.01$, * $p < 0.05$, post-hoc Tukey–Kramer test following two-way analyses of variance on the ranks. The numbers of analysed pairs and ensembles are summarised in Supplementary Tables 4 and 5, respectively. Detailed statistics are shown in Supplementary Data 1, and source data are provided as a Source Data file.

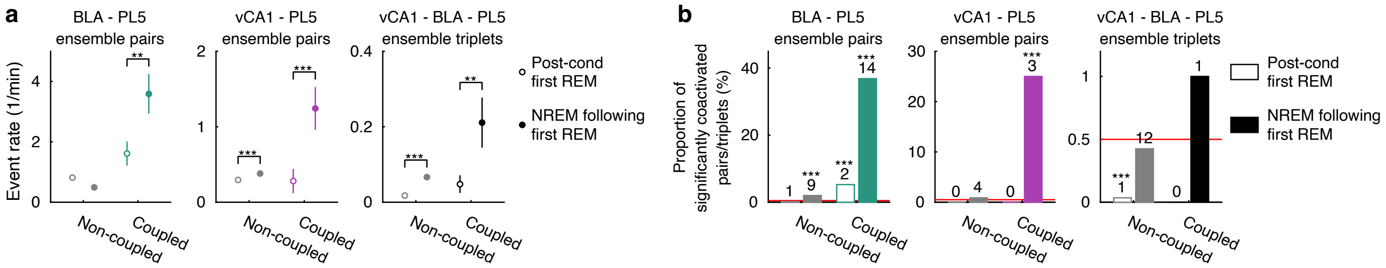

Supplementary Fig. 20 Significant ensemble coactivation in NREM following the first REM in post-conditioning homecage sessions.

a Mean coactivation/triple-activation event rates in post-conditioning (post-cond) first REM epochs (open) and first NREM epochs following the first REM (filled). Error bars indicate standard errors of the mean. *** $p < 0.001$, ** $p<0.01$, Wilcoxon rank sum test.

b Proportion of ensemble pairs/triplets that showing significant coactivation/triple-activation in post-conditioning first REM (open) and following NREM (filled). The numbers of pairs/triplets are superimposed on top of the bars. The horizontal red lines indicate the chance level (0.5%). *** $p < 0.001$, χ^2 test.

The numbers of analysed pairs and triplets are summarised in Supplementary Table 4. Detailed statistics are shown in Supplementary Data 1, and source data are provided as a Source Data file.

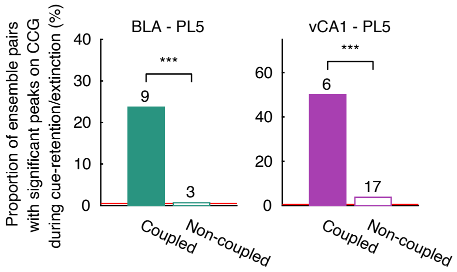

Supplementary Fig. 21 Ensemble coactivation that occurred during the post-conditioning NREM reappeared during cue-retention/extinction sessions.

The proportion of ensemble pairs that had significant CCG peaks during cue-retention/extinction sessions was significantly higher in coupled-ensemble-pairs (filled bars) than in non-coupled pairs (open bars) in both BLA–PL5 (left panel) and vCA1–PL5 (right panel) ensemble pairs. Note that coupled/non-coupled-ensemble-pairs were classified as pairs with/without significant CCG peaks during post-conditioning NREM, respectively. The number of ensembles are superimposed on the bars. *** $p < 0.001$, Fisher's exact test. Red horizontal bars indicate the chance level (0.5%). The numbers of analysed pairs are summarised in Supplementary Table 4. Detailed statistics are shown in Supplementary Data 1, and source data are provided as a Source Data file.

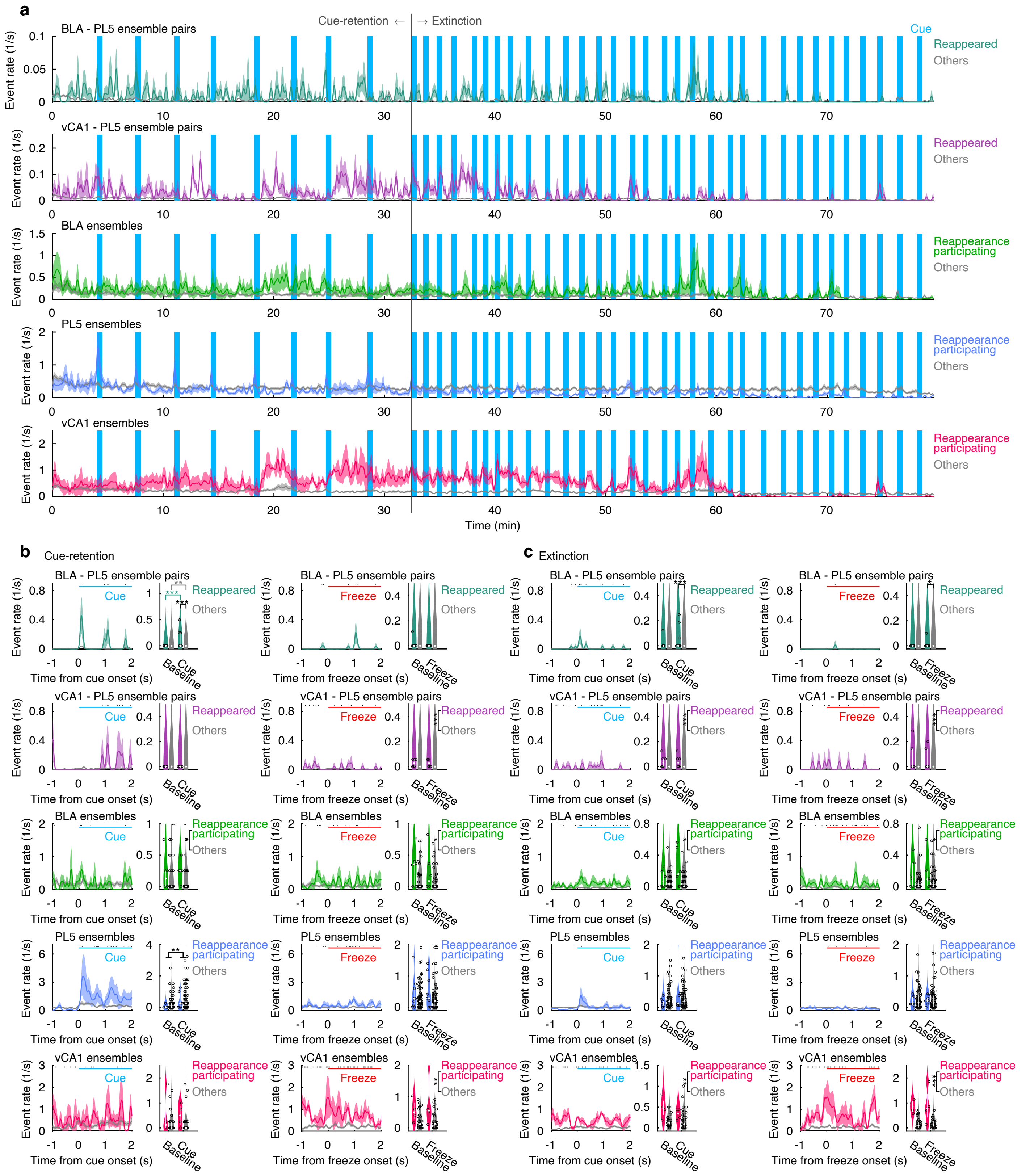

Supplementary Fig. 22 Time course of cue and freeze modulations of ensemble activation and coactivation event rates during cue-retention/extinction.

a Mean coactivation event rates (top two plots) and ensemble activation event rates (bottom three plots) calculated using 0.1 s bins and smoothed with Gaussian kernel (σ = 5 s). On the top two plots, coloured and grey lines indicate reappeared-ensemble-pairs and other ensemble pairs, respectively. On the bottom three plots, coloured and grey lines show ensembles participating in reappeared-ensemble-pairs and others, respectively. Lines and shaded areas represent means and standard errors of the mean (SEMs), respectively.

b, c Cue (left) and freeze (right) onset-triggered average of ensemble pair coactivation event rates (top two rows) and ensemble activation rates (bottom three rows) during cue-retention (**b**) and extinction (**c**). Violin plots on the right represent mean event rates in 0.05–0.55 s periods prior to and following triggers (baseline and cue/freeze, respectively). Shaded areas on the left panels represent SEMs. On the right panels, white dots and lines represent medians and upper/lower quartiles, respectively. Individual data points are shown with open circles except for ensemble-pairs that did not reappear (others). On the left panels, periods with significant differences between reappeared-ensemble-pairs and other ensemble pairs or between ensembles participating in reappeared-ensemble-pairs and other ensembles are indicated with black ticks on the top ($p < 0.05$, WSR-test). *** $p < 0.001$, ** $p < 0.01$, * $p < 0.05$, post-hoc Tukey–Kramer test following two-way analysis of variance on the ranks.

The numbers of ensembles participating in reappeared-ensemble-pairs and those of reappeared-ensemble-pairs and are summarised in Supplementary Tables 5 and 6, respectively. Detailed statistics are shown in Supplementary Data 1, and source data are provided as a Source Data file.

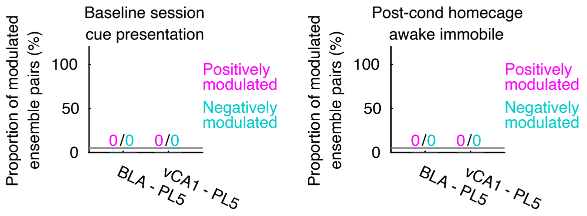

Supplementary Fig. 23 Ensemble coactivations during wakefulness are not modulated by sensory inputs or immobility.

Among the reappeared-ensemble-pairs (coupled-ensemble-pairs that are coactivated significantly during cue-retention/extinction sessions; n = 9 for BLA–PL5 pairs and n = 6 for vCA1–PL5 pairs), none were significantly modulated by cue presentation in baseline sessions (left) or by awake immobility in post-conditioning (post-cond) homecage sessions (right). Source data are provided as a Source Data file.

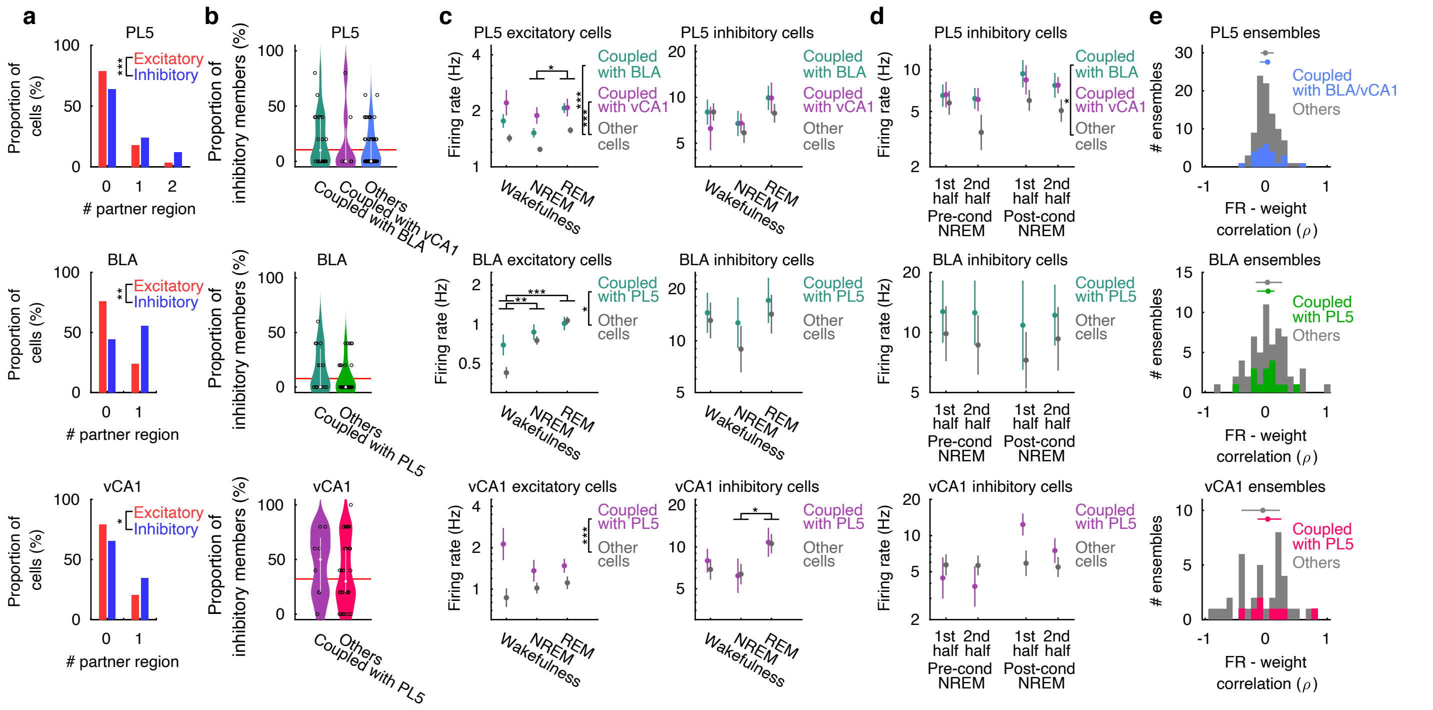

Supplementary Fig. 24 Firing properties of ensemble-coactivation-contributing cells.

a Histograms showing the number of paired regions per cell in the PL5, BLA, and vCA1. *** $p < 0.001$, ** $p < 0.01$, * $p < 0.05$, Fisher's exact test.

b Violin plots showing proportions of inhibitory cells among all member cells in the PL5, BLA, and vCA1. Horizontal red bars indicate the proportion of inhibitory cells over all recorded neurons in the region of interest. White dots and lines indicate medians and upper/lower quartiles, respectively. Individual data points are superimposed with open circles. No significant difference was detected among ensemble types ($p > 0.06$ for all, Wilcoxon rank-sum [WRS] test or Kruskal–Wallis test).

c Mean firing rates of ensemble-coactivation-contributing excitatory/inhibitory cells and other excitatory/inhibitory cells during wakefulness, NREM, and REM. The wakefulness/NREM/REM periods detected in all five homecage sessions were concatenated to calculate mean firing rates for each brain state. Cells not coupled with the PL5, BLA, or vCA1 are indicated as 'other cells'. The error bars indicate the standard errors of the mean (SEMs). *** $p < 0.001$, ** $p < 0.01$, * $p < 0.05$, post-hoc Tukey–Kramer (TK) test following two-way analysis of variance (ANOVA) on logarithms of the data.

d Mean firing rates of inhibitory cells during NREM in the 1st and 2nd halves of pre- and post-conditioning (cond) homecage sessions. The error bars indicate the SEMs. * $p < 0.05$, post-hoc TK test following two-way ANOVA on logarithms of the data.

e Histograms of Spearman's rank-order correlation coefficients (ρ) between the firing rates (FR) of excitatory cells versus the weights of projection vectors. Coactivation-participating ensembles (colour) and other ensembles (grey) are shown separately, and no significant differences were detected (n = 15/45, 8/30, 25/68 coactivation-participating/other ensembles in BLA, vCA1, and PL5, respectively; $p > 0.5$ for all, WRS-test). In addition, the median of correlations between FR and weights were not significantly different from zero ($\rho = 0.04 \pm 0.04$ / -0.05 ± 0.07 / 0.01 ± 0.02 [mean ± SEM] for BLA/vCA1/PL5, respectively; n = 60 / 38 / 93 ensembles, $p > 0.3$ for all, Wilcoxon signed rank test). The median and quartiles of the distribution are shown at the top of the plot.

The numbers of analysed cells in **(a)** and **(c, d)** are summarised in Supplementary Table 1 and 8, respectively, and those of analysed ensembles in **(b)** are summarised in Supplementary Table 7. Detailed statistics are shown in Supplementary Data 1, and source data are provided as a Source Data file.

Supplementary Table 1 Number of recorded cells.

	Brain region										
Rat name	vCA1	BLA	PL5	vCA3	vSub	LA	CeA	PL2/3	Pir	STIA	Other
Achel	–	3, 2, 0	–	3, 2, 1	–	–	0, 0, 0	6, 1, 1	–	2, 0, 1	1, 0, 2
Booyah	–	18, 1, 0	0, 0, 0	0, 0, 0	–	–	–	40, 4, 0	–	–	2, 0, 0
Chimay	–	0, 0, 0	0, 0, 0	26, 13, 1	–	16, 1, 0	–	7, 1, 0	–	2, 0, 0	0, 1, 1
Duvel	4, 0, 2	16, 0, 3	–	–	–	–	–	15, 1, 3	–	15, 1, 1	0, 1, 1
Estrella	–	12, 2, 0	0, 0, 0	–	8, 2, 0	–	–	29, 1, 0	5, 1, 4	–	0, 0, 0
Feuillien	–	–	0, 0, 0	–	13, 1, 0	5, 0, 0	2, 0, 0	3, 0, 0	–	1, 0, 0	0, 1, 0
Guiness	–	11, 0, 0	–	0, 0, 0	17, 6, 1	0, 0, 0	0, 0, 0	1, 0, 0	–	7, 1, 0	0, 0, 0
Hoegaarden	28, 6, 1	63, 4, 0	41, 6, 4	0, 0, 0	–	–	–	–	–	0, 0, 0	1, 1, 0
Innis	13, 4, 0	8, 2, 0	56, 9, 2	0, 1, 0	–	0, 0, 0	2, 0, 1	–	–	–	1, 1, 0
Jever	–	7, 0, 1	43, 2, 2	–	3, 2, 0	–	–	–	–	0, 0, 1	1, 1, 0
Karmeliet	20, 6, 0	–	74, 7, 0	1, 0, 0	–	17, 0, 0	22, 2, 2	–	–	–	1, 1, 0
Leffe	5, 1, 0	5, 0, 1	48, 3, 1	–	–	0, 0, 0	–	–	–	–	2, 1, 0
Maredsous	6, 5, 0	20, 3, 0	33, 6, 2	–	4, 0, 0	1, 0, 0	–	–	–	0, 0, 1	0, 1, 0
Nostrum	10, 17, 0	33, 4, 0	60, 9, 0	–	–	0, 0, 0	–	–	–	5, 0, 0	1, 1, 0
Oberon	6, 7, 2	13, 0, 0	63, 8, 0	–	–	–	1, 0, 0	–	–	1, 1, 2	0, 0, 0
Total	92, 46, 5	209, 18, 5	418, 50, 11	30, 16, 2	45, 11, 1	39, 1, 0	27, 2, 3	101, 8, 4	5, 1, 4	33, 3, 6	10, 10, 4

Numbers of well-isolated excitatory, inhibitory, and non-classified cells in each brain region of individual rats. The numbers include cells with positive spikes.

vCA1, ventral hippocampus CA1 region; BLA, basolateral nucleus of the amygdala; PL5, prelimbic cortex layer 5; vCA3, ventral hippocampus CA3 region; vSub, ventral subiculum; LA, lateral nucleus of the amygdala; CeA, central nucleus of the amygdala; PL2/3, prelimbic cortex layer 2/3; Pir, pyriform cortex; STIA, bed nucleus of the stria terminalis intra-amygdaloid division.

Supplementary Table 2 Number of identified cell ensembles.

	Brain region										
Rat name	vCA1	BLA	PL5	vCA3	vSub	LA	CeA	PL2/3	Pir	STIA	Other
Achel	–	2	–	2	–	–	0	2	–	0	0
Booyah	–	6	0	0	–	–	–	8	–	–	0
Chimay	–	0	0	10	–	6	–	1	–	0	0
Duvel	1	3	–	–	–	–	–	5	–	4	0
Estrella	–	4	0	–	4	–	–	7	2	–	0
Feuillien	–	–	0	–	4	1	0	0	–	0	0
Guiness	–	2	–	0	7	0	0	0	–	2	0
Hoegaarden	8	17	10	0	–	–	–	–	–	0	0
Innis	5	2	12	0	–	0	0	–	–	–	0
Jever	–	2	8	–	1	–	–	–	–	0	0
Karmeliet	7	–	15	0	–	4	4	–	–	–	0
Leffe	2	2	8	–	–	0	–	–	–	–	0
Maredsous	3	7	11	–	0	0	–	–	–	0	0
Nostrum	7	9	14	–	–	0	–	–	–	2	0
Oberon	5	4	15	–	–	–	0	–	–	0	0
Total	38	60	93	12	16	11	4	23	2	8	0

Number of identified cell ensembles in each brain region of individual rats. Ensembles were defined during conditioning sessions.

BLA, basolateral nucleus of the amygdala; CeA, central nucleus of the amygdala; LA, lateral nucleus of the amygdala; Pir, pyriform cortex; PL2/3, prelimbic cortex layer 2/3; PL5, prelimbic cortex layer 5; STIA, bed nucleus of the stria terminalis intra-amygdaloid division; vCA1, ventral hippocampus CA1 region; vCA3, ventral hippocampus CA3 region; vSub, ventral subiculum.

Supplementary Table 3 Number of inter-regional ensemble pairs.

Region pair	Number of pairs	Coupled pairs	Inverse-coupled pairs	Number of examined rats
BLA - PL5	489	38 (7.8%)	10 (2.0%)	7
vCA1 - PL5	467	12 (2.6%)	3 (0.6%)	7
vCA1 - BLA	257	4 (1.6%)	1 (0.4%)	7
BLA - Pir	8	0 (0.0%)	0 (0.0%)	1
BLA - PL2/3	95	3 (3.2%)	2 (2.1%)	4
BLA - STIA	34	1 (2.9%)	1 (2.9%)	3
BLA - vCA3	4	0 (0.0%)	0 (0.0%)	1
BLA - vSub	32	0 (0.0%)	0 (0.0%)	3
vCA1 - CeA	28	0 (0.0%)	0 (0.0%)	1
vCA1 - LA	28	0 (0.0%)	0 (0.0%)	1
vCA1 - PL2/3	5	0 (0.0%)	0 (0.0%)	1
vCA1 - STIA	18	0 (0.0%)	0 (0.0%)	2
PL5 - STIA	28	0 (0.0%)	0 (0.0%)	1
PL5 - CeA	60	0 (0.0%)	0 (0.0%)	1
PL5 - LA	60	0 (0.0%)	0 (0.0%)	1
PL5 - vSub	8	0 (0.0%)	0 (0.0%)	1
vCA3 - LA	60	2 (3.3%)	3 (5.0%)	1
vCA3 - PL2/3	14	0 (0.0%)	0 (0.0%)	2
vSub - LA	4	0 (0.0%)	0 (0.0%)	1
vSub - Pir	8	0 (0.0%)	0 (0.0%)	1
vSub - PL2/3	28	0 (0.0%)	0 (0.0%)	1
vSub - STIA	14	1 (7.1%)	2 (14.3%)	1
LA - CeA	16	0 (0.0%)	0 (0.0%)	1
LA - PL2/3	6	0 (0.0%)	0 (0.0%)	1
PL2/3 - Pir	14	0 (0.0%)	0 (0.0%)	1
PL2/3 - STIA	20	1 (5.0%)	1 (5.0%)	1

Number of simultaneously recorded inter-regional ensemble pairs and the number and proportion of coupled- and inverse-coupled-ensemble-pairs. The chance levels for the proportions of coupled- and inverse-coupled-ensemble-pairs are 0.5%. The rightmost column shows the number of rats in which coactivation of ensembles in the corresponding region pair was examined.

BLA, basolateral nucleus of the amygdala; CeA, central nucleus of the amygdala; LA, lateral nucleus of the amygdala; Pir, pyriform cortex; PL2/3, prelimbic cortex layer 2/3; PL5, prelimbic cortex layer 5; STIA, bed nucleus of the stria terminalis intra-amygdaloid division; vCA1, ventral hippocampus CA1 region; vCA3, ventral hippocampus CA3 region; vSub, ventral subiculum.

Supplementary Table 4 Number of coupled-ensemble-pairs and coupled-ensemble-triplets in each rat.

	Ensemble pairs				Ensemble triplets
Rat name	BLA - PL5	vCA1 - PL5	vCA1 - BLA	Others	BLA - vCA1 - PL5
Achel	–	–	–	0/12	–
Booyah	–	–	–	2/48	–
Chimay	–	–	–	2/76	–
Duvel	–	–	0/3	2/56	–
Estrella	–	–	–	0/102	–
Feuillien	–	–	–	0/4	–
Guiness	–	–	–	2/32	–
Hoegaarden	11/170	1/80	1/136	–	16/1360
Innis	3/24	7/60	0/10	–	7/120
Jever	0/16	–	–	0/10	–
Karmeliet	–	2/105	–	0/192	–
Leffe	0/16	0/16	0/4	–	2/32
Maredsous	5/77	0/33	2/21	–	3/231
Nostrum	17/126	2/98	1/63	0/60	64/882
Oberon	2/60	0/75	0/20	–	8/300
Sum	38/489	12/467	4/257	8/592	100/2925

(Left; ensemble pairs) Number of coupled and total ensemble pairs among BLA, vCA1, PL5, and other region pairs. (Right; ensemble triplets) Number of coupled and total ensemble triplets across BLA, vCA1, and PL5.

Supplementary Table 5 Number of coactivation-, triplet-, and reappearance-participating ensembles.

	Coactivation-participating / others	Triplet-participating / others	Reappearance-participating / others
Ensembles in BLA	15 / 45	20 / 40	6 / 54
Ensembles in vCA1	8 / 30	21 / 17	4 / 34
Ensembles in PL5	25 / 68	38 / 55	9 / 84

Number of coactivation-participating-ensembles (i.e., ensembles that participated in at least one coupled-ensemble-pair), triplet-participating-ensembles (i.e., ensembles that participated in at least one coupled-ensemble-triplet), reappearance-participating-ensembles (i.e., ensembles that participated in at least one reappeared-ensemble-pair), and other ensembles/triplets in the BLA, vCA1, and PL5.

Supplementary Table 6 Number of reappeared-ensemble-pairs.

	Reappeared ensemble pairs	Other ensemble pairs
BLA - PL5 ensemble pairs	9	480
vCA1 - PL5 ensemble pairs	6	461

Number of reappeared-ensemble-pairs (ensemble pairs with significant CCG peaks both in post-conditioning NREM and cue-retention/extinction session) and other ensemble pairs for the BLA–PL5 and vCA1–PL5 ensemble pairs. BLA–vCA1 ensemble pairs were not examined.

Supplementary Table 7 Number of coactivation-participating-ensembles.

	Coupled with BLA	Coupled with vCA1	Coupled with PL5	Other ensembles
Ensembles in BLA	N.A.	N.A.	15	45
Ensembles in vCA1	N.A.	N.A.	8	30
Ensembles in PL5	22	7	N.A.	68

BLA–vCA1 ensemble pairs were not examined. Ensembles not coupled with BLA, vCA1, or PL5 are shown as other ensembles. NA, not applicable.

Supplementary Table 8 Number of ensemble-coactivation-contributing cells.

	Coupled with BLA	Coupled with vCA1	Coupled with PL5	Other cells
Cells in BLA	N.A.	N.A.	50 / 10 / 0	159 / 8 / 5
Cells in vCA1	N.A.	N.A.	19 / 16 / 0	73 / 30 / 5
Cells in PL5	75 / 18 / 3	28 / 6 / 1	N.A.	329 / 32 / 7

Number of excitatory/inhibitory/non-classified ensemble-coactivation-contributing cells (members of ensembles coupled with other brain regions). BLA/vCA1 cells coupled with vCA1/BLA were not examined. Cells not coupled with BLA, vCA1, or PL5 are shown as other cells. NA, not applicable.
