# Supplementary material for: De novo inter-regional coactivations of preconfigured local ensembles support memory
Source: Nat Commun. 2022 Mar 11;13:1272. doi: 10.1038/s41467-022-28929-x (PMC8917150; doi:10.1038/s41467-022-28929-x)
Supplement: Supplementary file 3 — Description of Additional Supplementary Files [file 41467_2022_28929_MOESM3_ESM.pdf]

Inventory of Supporting Information

Supplementary Information

A PDF file that contains 24 supplementary figures, 8 supplementary tables, and their legends.

Supplementary Data

An Excel spreadsheet that contains details of the statistics.

Source Data File

A zipped folder that contains source data of figures and supplementary figures (111 text files in total).

Reporting summary
